# Triplane osteotomy combined with talar non-weight-bearing area autologous osteochondral transplantation for osteochondral lesions of the talus

**DOI:** 10.1186/s12891-022-05043-z

**Published:** 2022-01-22

**Authors:** Yan Zhang, Jing-qi Liang, Xiao-dong Wen, Pei-long Liu, Jun Lu, Hong-mou Zhao

**Affiliations:** Department of Foot and Ankle Surgery, Honghui Hospital, Xi’an Jiaotong University, 710054, Xi’an, Shaanxi Province China

**Keywords:** Talus, Medial malleolar osteotomy, Donor, Autograft, Osteochondral transplantation, Osteochondral lesions

## Abstract

**Background:**

Traditional medial malleolar osteotomy combined with autologous osteochondral transplantation (AOT) is mostly used in the treatment of osteochondral lesions of the talus (OLTs), but with high osteotomy and donor site complications. We hypothesis a new triplane medial malleolar osteotomy combined with AOT from non-weight-bearing area of the talus could be a promising choice for OLTs.

**Methods:**

We reviewed all the symptomatic OLTs patients who received AOT with triplane osteotomy of the medial malleolus between September 2015 and December 2017 in our department. According to the inclusion and exclusion criteria, 23 patients (23 ankles), including 14 males and 9 females, were included in the study. The mean age was 35.6 years. The mean size of the lesion area was 141.5 mm^2^. According Ferkel’s classification, including 5 type I, 11 typeIIa and 7 typeIIb. The visual analog scale (VAS) for pain during walking and the American Orthopaedic Foot and Ankle Society (AOFAS) ankle-hindfoot score were used for the pre- and postoperative evaluations. In addition, the incorporation of the grafts was assessed by computed tomography (CT).

**Results:**

All patients had a minimum follow-up of 22 months, with an average of 37.1 months. The mean time from osteotomy to full weight-bearing activity was 8.1 ± 2.3 weeks (range, 5–12 weeks). The mean VAS score improved from 5.6 ± 0.7 preoperatively to 0.7 ± 1.0 postoperatively (*P* < 0.01). The AOFAS ankle-hindfoot score improved significantly in all domains (P < 0.01). Twenty-one patients returned to sport at their previous level, and 2 returned at a lower level compared with preinjury (mean return to play, 7.4 months). According to CT, the medial malleolus recovered in all patients, and the graft was incorporated well. One patient suffered from flexor hallucis longus tendon discomfort due to internal fixation screw irritation posteromedial to the ankle. The general complication rate was 4.3% (1/23).

**Conclusions:**

These results indicate that AOT combined with medial malleolus triplane osteotomy maybe a viable option for OLTs. Patients could perform weight-bearing exercise and return to sport as early as possible, with a lower rate of complications at the osteotomy site and donor site. However, the large sample well-designed prospective comparative studies are still needed.

## Background

Osteochondral lesions of the talus (OLTs) are one of the most common sports injuries of the ankle joint. In addition, 50–73% of these lesions are associated with acute ankle injury events [1, 2], and sports participants are a susceptible population. OLTs is a kind of progressive disease, and may cause irreversible damage to the ankle joint and affect the quality of life with time delayed [3]. The treatment of symptomatic OLTs is difficult because of the poor blood supply of the talus. Conservative treatments for symptomatic OLTs include nonsteroidal drugs, sodium hyaluronate injection, physiotherapy, etc. However, a review reported that the success rate of conservative treatment was approximately 45% [4]. If nonoperative treatment fails, alternative surgical interventions include arthroscopic debridement and bone marrow stimulation (BMS), autologous osteochondral transplantation (AOT), allogeneic osteochondral transplantation, autologous or juvenile chondrocyte implantation, biological agents, and Hemi CAP® prosthetic implantation [5, 6]. Among these methods, arthroscopic procedures are mainly used for the cartilage lesions, and if the damage is deeper than 6 mm, AOT is mostly used to solve the cartilage and bony problems [7].

**Fig. 1:**
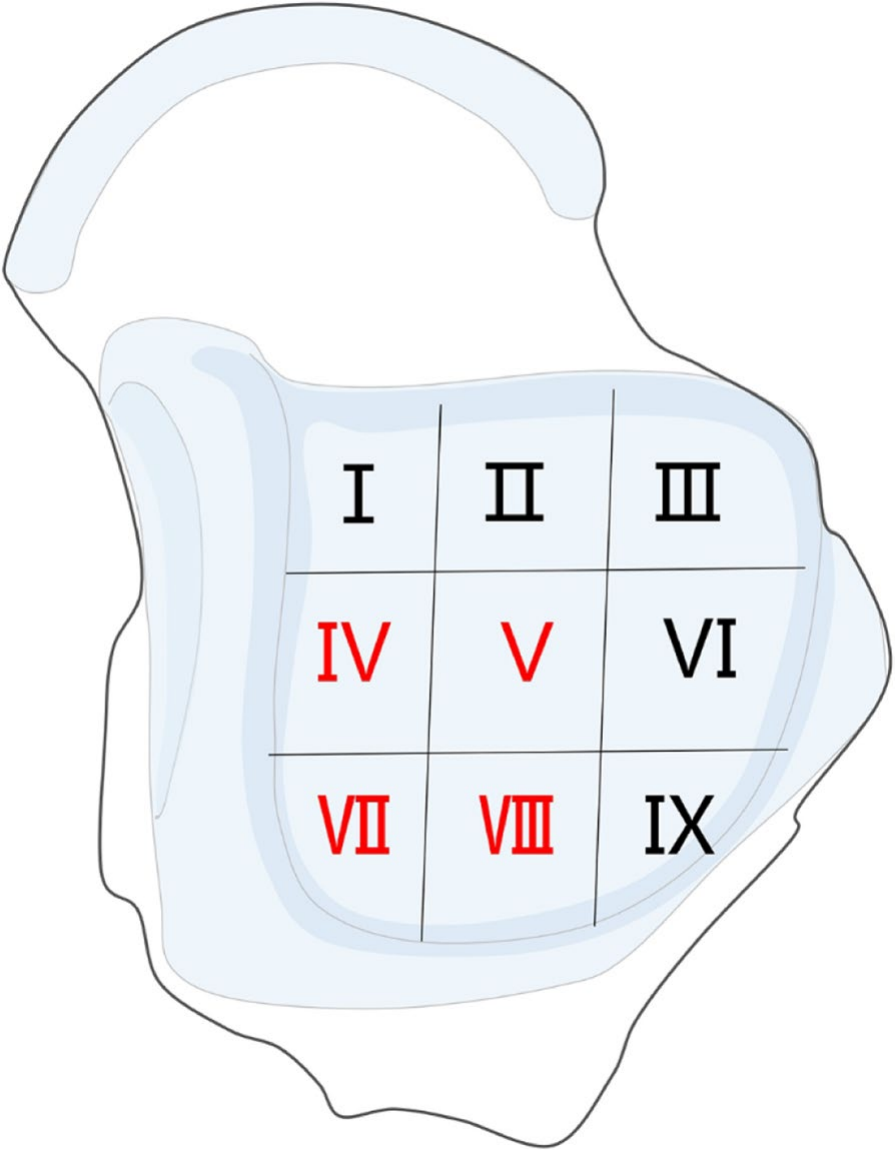
OLTs are common in the fourth to fifth and seventh to eighth regions

Nguyen et al. [8] found that 78% of OLTs were located in the posteromedial area of the talus dome, i.e., areas IV-V and areas IV-VIII according to the “Division of the talus” [9] (Fig. 1). OLTs in these areas always require medial malleolar osteotomy to fully expose the lesions. However, a study [10] reported that the malunion rate after traditional medial malleolar oblique osteotomy was as high as 30%, and approximately 60% of patients had residual pain and complaints at the osteotomy site, which seriously limited the outcomes of this procedure.

To reduce the complications related to traditional medial malleolar osteotomy, we designed a new triplane osteotomy method. In addition, the non-weight-bearing area of the ipsilateral talus was used as the donor site for AOT. We hypothesized that this new medial malleolar triplane osteotomy method would reduce osteotomy-related complications, and AOT from the non-weight-bearing area of the ipsilateral talus could achieve satisfactory clinical outcomes for OLTs.

## Methods

The study was approved by the local institutional ethics committee. The authors retrospectively studied the clinical and radiological outcomes of OLTs patients who underwent medial malleolar triplane osteotomy with non-weight-bearing autografts between September 2015 and November 2017. The inclusion criteria were as follows: (1) adults older than 18 years of age; (2) a confirmed diagnosis of OLTs concomitant with a cystic depth greater than 6 mm by computed tomography (CT); (3) lesions located in areas IV, V, VI, VIII of the talus dome; (4) ineffective conservative treatment for more than 6 months; and (5) at least 22 months of follow-up. The exclusion criteria were as follows: (1) subchondral defect greater than 154 mm^2^ after debridement in the operation; (2) fracture malunion or malalignment of the lower limb; (3) ankle degeneration; (4) ankle joint instability; (5) a history of medial malleolar fracture or osteotomy; (6) revision surgery; and (7) poor general health condition.

A total of 51 OLT patients treated operatively were initially identified. According to the inclusion and exclusion criteria, 23 patients (14 males and 9 females) were included in the current study. The average age was 31.4 ± 7.2 (range, 19–46) years, and the mean body mass index (BMI) was 25.1 ± 3.7 (range, 19–33). Among the 23 ankles, surgery was performed on the left ankle in 13 patients (57%) and on the right ankle in 10 patients (43%), and the average duration of symptoms was 12.7 ± 6.3 (range, 6–33) months. The injury causes were ankle sprains (57%) and fractures (13%), and the injury cause could not be determined in 7 patients. For each patient, plain radiography, CT, and magnetic resonance imaging (MRI) of the ankle joint were obtained, and the diagnosis of OLTs was confirmed. According to the “9-zone anatomic grid system”, 19 lesions (83%) were located in zone 4, 1 lesion (4%) was located in zone 5, and 3 lesions (13%) were located in zone 7. In the current study, OLTs were classified by Ferkel’s classification [11] based on CT scans. Among the OLTs, 5 OLTs were type I, 11 OLTs were typeIIa and 7 OLTs were typeIIb. The average area of the lesion was 113.2 ± 28.1 (range, 63.6–153.9) mm^2^, and the mean depth of the cyst was 11.0 ± 2.5 (range, 7.0–16.0) mm after debridement during the operation (Table 1).

**Table 1:** Demographic data of OLT patients

Cases	Sex	Age (yr)	BMI	Side	Etiology	Duration (mo)	Classification	Location	Area of lesion (mm^**2**^)	Depth of lesion (mm)
1	F	29	25	R	Sprain	11	IIa	7	78.5	7
2	M	32	26	L	Fracture	20	IIa	4	113.1	13
3	F	35	22	R	Sprain	12	I	4	132.7	8
4	M	36	30	L	Unknown	8	IIa	4	78.5	16
5	M	44	25	L	Sprain	19	IIb	4	113.1	9
6	M	30	24	R	Fracture	12	IIb	4	132.7	10
7	F	31	26	R	Sprain	6	IIa	4	153.9	9
8	M	37	27	L	Unknown	9	IIb	7	132.7	11
9	M	42	23	L	Unknown	10	IIa	4	113.1	13
10	F	31	29	L	Sprain	7	I	4	113.1	11
11	M	27	28	R	Sprain	33	IIa	4	78.5	9
12	M	20	30	R	Sprain	21	IIa	4	153.9	14
13	F	31	21	L	Unknown	9	IIb	4	63.6	10
14	F	29	23	L	Unknown	11	IIa	4	113.1	9
15	M	19	27	R	Sprain	6	I	4	78.5	7
16	M	27	21	L	Sprain	8	I	5	78.5	9
17	F	23	19	L	Unknown	13	IIa	4	153.9	13
18	M	24	22	R	Sprain	12	IIa	4	132.7	10
19	F	41	33	L	Sprain	9	IIb	4	132.7	14
20	M	30	24	L	Unknown	10	IIb	7	78.5	11
21	M	27	22	L	Sprain	14	IIa	4	113.1	15
22	F	46	30	R	Fracture	21	I	4	132.7	11
23	M	32	20	R	Sprain	12	IIb	4	132.7	13

### Surgical technique

All the operations were finished by the same surgeon (ZHM). Under general anesthesia, the patient was placed in a supine position with a tourniquet on the affected thigh. A 6 cm longitudinal curved incision was made from the anteromedial part of the inferior tibia and ended at the medial malleolar tip. The great saphenous vein was carefully located and protected. Medial malleolar triplane osteotomy method: First, a transverse osteotomy was performed in the anteromedial part of the ankle 2 cm above the tibial distal surface, with a depth of the anterior half of the tibia and a width of the medial half of the tibia. Second, a coronal osteotomy was performed along the midline of the medial malleolus. Third, a sagittal osteotomy was performed following the midline of the anterior tibia. Finally, the osteotomy block was carefully separated from the tibia with a thin osteotome. While the fragment was flipped downwards, the OLTs could be further visualized.

After the insertion of an appropriate recipient tube harvester, the unhealthy osteochondral plug was removed. The wall and base of the lesions were abraded and curetted down to the viable subchondral bone, and then a 1.0 mm K-wire was used to make a “microfracture” in the lesions. After that, the articular cartilage was reconstructed by a cylindrical autologous osteochondral plug taken from the medial non-weight-bearing area of the ipsilateral talus. If the recipient cavity was too loose so that the graft was at risk of detachment, the gap was filled with autogenous cancellous tissue, which was harvested from the distal tibial section after the medial malleolar osteotomy until stabilization. The donor bone defect was filled with an autologous bone graft with periosteum from the tibia by the same recipient tube harvester. The periosteum was flush with the surrounding cartilage surface. Especially note that when the column was harvesting from the medial side of distal tibia, the periosteum was protected to ensure that it does not separate from the cortical bone.

**Fig. 2:**
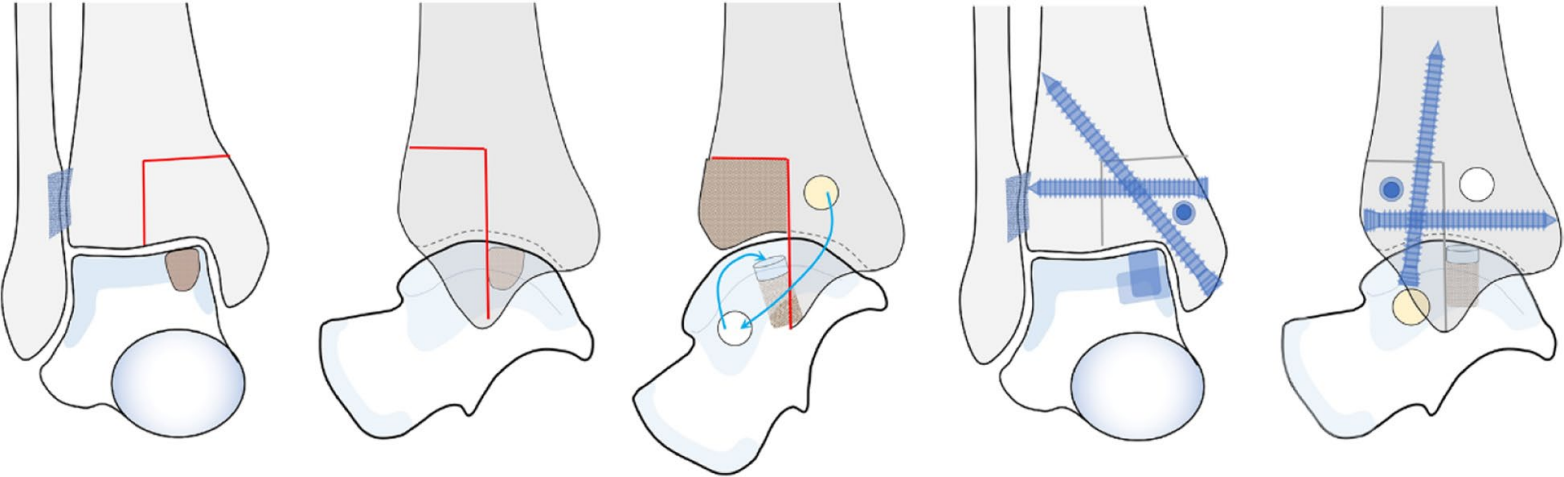
**A**-**B**: medial malleolar triplane osteotomy; **C**: AOT for OLTs from a non-weight-bearing area, and the donor defect was filled with a bone graft with periosteum from the tibia; **D**-**E**: medial malleolar osteotomy fixed with screws

**Fig. 3:**
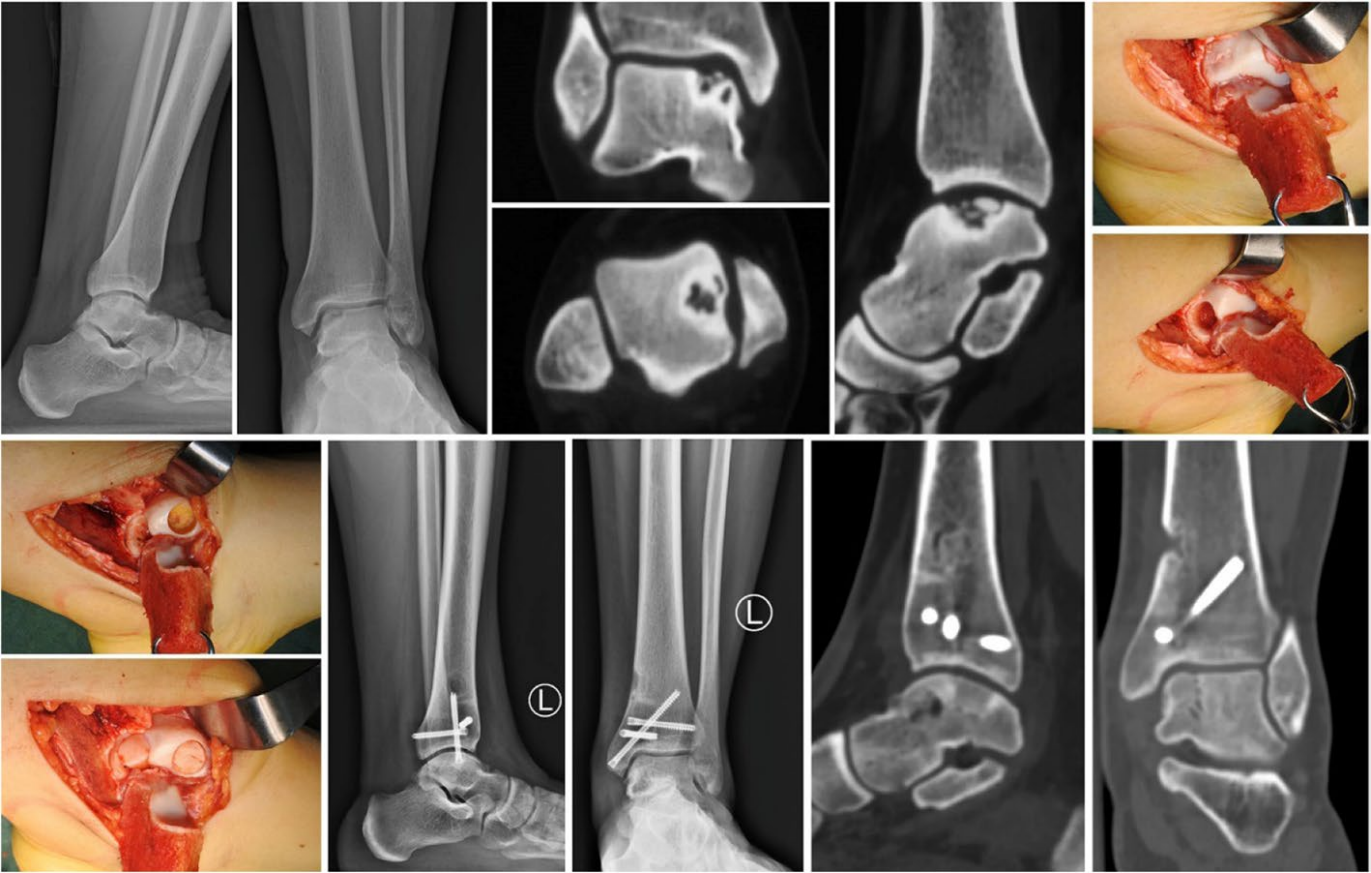
Case1, female, 31y, osteochondral lesions of left talus. **A**-**E**: preoperative imaging (X - ray and CT) supported the diagnosis; **F**-**G**: the lesion was exposed through the medial malleolus triplane osteotomy and debrided; **H**-**I**: AOT for OLTs from a non-weight-bearing area, and the donor defect was filled with a bone graft with periosteum from the tibia; **J**-**M**: at 37-month follow-up, imaging showed that the grafts had fused with the original bone in the talus, and the medial malleolus had achieved anatomic union

**Fig. 4:**
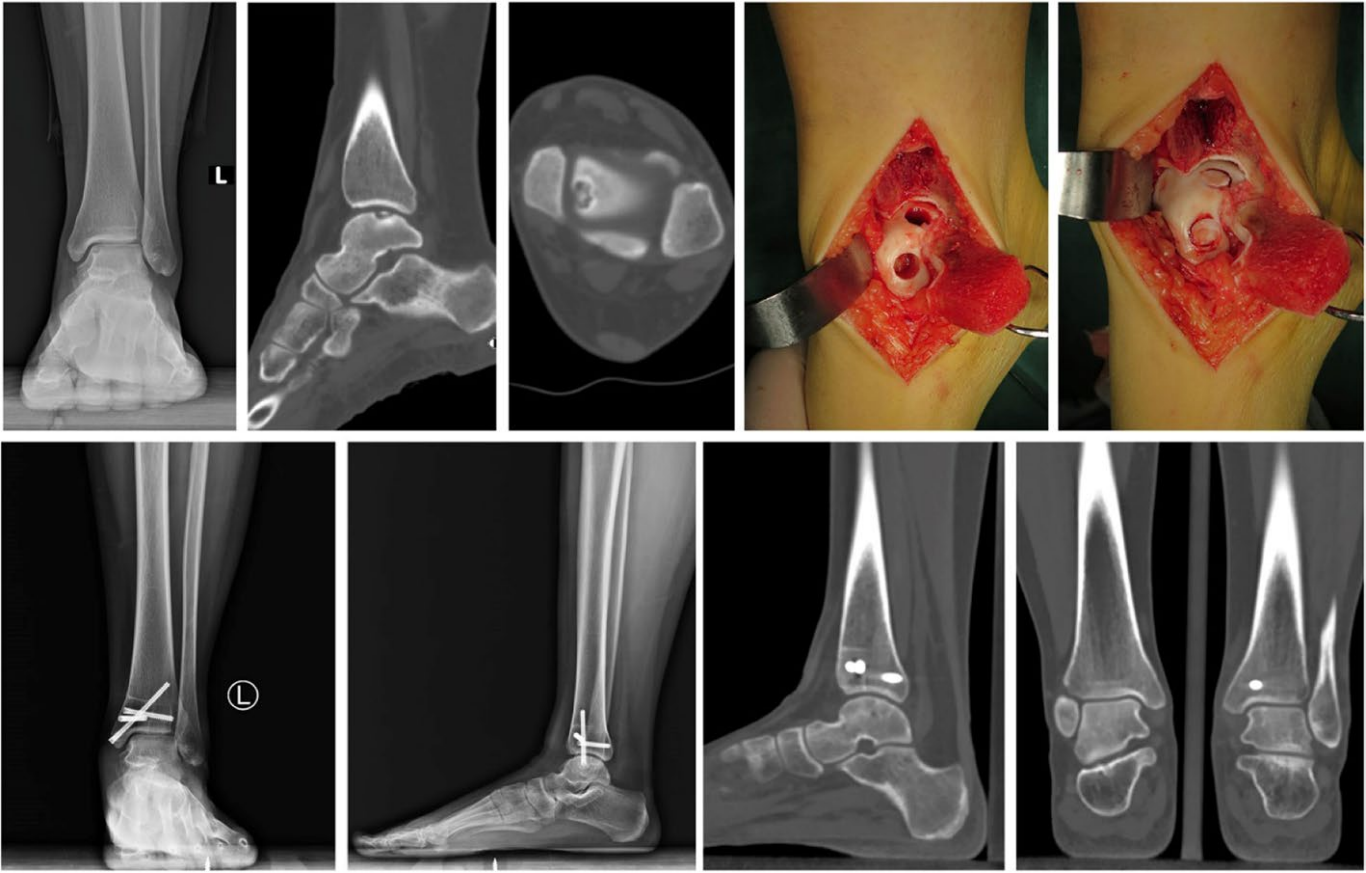
Case2, female, 41y, osteochondral lesions of left talus. **A**-**C**: preoperative imaging (X - ray and CT) supported the diagnosis; **D**-**E**: the lesion was exposed through the medial malleolus triplane osteotomy and AOT for OLTs from a non-weight-bearing area, and the donor defect was filled with a bone graft with periosteum from the tibia; **F**-**I**: at 1.5-year follow-up, imaging showed that the grafts had fused with the original bone in the talus, and the medial malleolus had achieved anatomic union

Finally, the medial malleolar osteotomy site was fixed with 3 screws. The surgical diagram was seen in Figs. 2, and the operation detail was seen in Figs. 3 and 4.

### Postoperative care

The ankle joints were wrapped in a bandage for 3 weeks. Patients were encouraged to begin passive range-of-motion exercises for the ankle joint on the 3rd day after the operation The patients were initially restricted to non-weight-bearing activities, and in the absence of rehabilitation, they wore a short leg cast for protection. The patients wore removable walking boots and started partial weight-bearing activities at the 4th week post operation. Full weight-bearing activities began when X-rays showed callus formation, which is evidence of bone healing at the site of the medial malleolar osteotomy.

### Clinical and radiographic evaluation

The clinical outcome was assessed using the American Orthopedic Foot and Ankle Society ankle-hindfoot (AOFAS-AH) score and the visual analog scale (VAS) score. To show the actual impact of pain on patients’ daily activities, we evaluated the VAS score after 1 km of walking. The AOFAS-AH and VAS scores were obtained both preoperatively and at the final follow-up. In addition, we recorded the operation time, postoperative complications, time of full weight-bearing activities and the ROM of the affected ankle joint at the final follow-up. X-rays and CT scans were acquired for all patients to assess the reduction and healing of the medial malleolar osteotomy site, the union of grafts in the talus and the position and length of internal screws. X-ray is necessary in every follow-up after operation. After 3 months postoperatively, CT was used for evaluation at each follow-up. The malunion of medial malleolus osteotomy was defined as medial malleolus incongruence with measurable proximal or medial displacement. Degenerative changes (including narrowing of joint space, formation of osteophyte and increase of subchondral bone density) of ankle were observed based on X-ray. All the included clinical and radiological measurements on were performed by two observers independently (WXD and LPL), and the mean of the two observers was used as the result.

### Statistical analysis

IBM SPSS 21.0 software (SPSS Inc., Chicago, Illinois) was used for statistical analysis. The Shapiro-Wilk test was used to test whether the data were normally distributed: if the data were normally distributed, Student’s t-test was conducted to evaluate changes in the AOFAS-AH score and VAS score; if the data were not normally distributed, a rank sum test was used. The level of statistical significance was set at α = 0.05.

## Results

The mean follow-up time was 37.1 ± 7.3 (range, 22–49) months. The mean time of operation was 69.4 ± 12.6 (range, 51–97) minutes. All medical malleolar osteotomy sites healed anatomically at a mean of 8.1 ± 2.3 (range, 5–12) weeks, with neither malunion nor nonunion. All the grafts in the talus were well fused according to CT scans until the final follow-up. One patient suffered from flexor hallucis longus tendon discomfort due to internal fixation screw irritation posteromedial to the ankle. The symptoms disappeared after the removal of the screw at 6 months post operation. In the current study, the general complication rate was 4.3% (1/23). At the last follow-up, the mean angles of affected ankle plantar flexion and dorsiflexion were 44.7° and 12.1°, respectively. All patients were satisfied with the pain relief. The mean VAS score improved from 5.6 ± 0.7 (range, 4–7) to 0.7 ± 1.0 (range, 0–3). The mean AOFAS-AH score improved from 56.0 ± 9.5 (range, 40–78) before the operation to 93.8 ± 6.6 (range, 83–100) at the final follow-up (*p* < 0.01) (Table 2). In addition, typical cases are shown below (Figs. 3 and 4).

**Table 2:** Functional outcomes of AOT for OLTs

Time	AOFAS-AH score				VAS score (1 km)
	Pain	Function	Alignment	Total	
Preoperation	21.7 ± 3.9	24.3 ± 6.3	10	56.0 ± 9.5	5.6 ± 0.7
Postoperation	35.7 ± 5.1	48.1 ± 1.9	10	93.8 ± 6.6	0.7 ± 1.0
t	−13.371	−22.463	–	−24.835	33.631
p	0.000	0.000	–	0.000	0.000

## Discussion

The most important finding of this study is that medial malleolar triplane osteotomy with AOT from the non-weight-bearing area of the talus for OLTs achieved significant pain reduction and satisfactory ankle function with few complications after a mean follow-up of more than 3 years.

Throughout evolution, to satisfy the needs of flexible ankle joint movement in human upright walking and activities, the talus, located in the center of the ankle, has gradually acquired a unique anatomy in that the majority of its surface (approximately 60%) is covered with articular cartilage [12], and there is little soft tissue (including nutritional arteries) attachment in the body of the talus. On the other hand, the vessel spreading in the talus has failed to achieve a rich blood supply, forming a “watershed” of blood supply between single areas [13]. This anatomical limitation has been implicated in the high risk of posttraumatic osteonecrosis and OLTs [5, 13]. Nondisplaced or mild symptomatic OLTs are often initially treated with a nonoperative approach. Conservative treatments of OLTs include activity modification, protected weight-bearing, rehabilitation, bracing, and nonsteroidal anti-inflammatory drugs. Clinically, a systematic review by Verhagen [14] et al. showed that approximately 45% of patients with OLTs reported successful outcomes with conservative treatment. It is worth noting that the success of nonsurgical treatment for OLTs is mostly in the pediatric population, but it is limited in the adult population, which may be attributable to the relatively active repair ability of articular hyaline cartilage in young people. At present, there is no unified standard for surgical indications for OLTs. It is generally believed that patients with small OLTs less than 15 mm in diameter or less than 150 mm^2^ in area can obtain satisfactory clinical effects through BMS [5, 15], whereas patients with large OLTs or failed bone marrow stimulation should choose replacement procedures [5, 16]. A previous study [17] showed that the main pathway of BMS for OLTs is through a series of inflammatory reactions and cell differentiation to form new scar tissue (mainly type I collagen) to repair the talus defect area. However, some studies [18, 19] that used MRI to evaluate the imaging results of BMS in the treatment of OLTs found that the lesions could not be completely filled, the quality of scar tissue involved in the repair of articular surface was poor, and it was difficult to achieve complete fusion with primary cartilage. This may be related to the fact that the osteocytes under the cartilage of OLTs are not filled with bone after BMS treatment, resulting in the lack of effective physical support and nutritional supply for the surface tissue. Therefore, we believe that the limitation of osteochondral transplantation is not simply determined by the size of the lesion. In other words, even if the area of an articular cartilage lesion is relatively large (> 150 mm^2^) and there is no cystic change or the cyst is very shallow, the use of BMS can also obtain sufficient scar tissue filling and repair. In contrast, for OLTs with significant cystic lesions, even if the area of cartilage lesions is not large, the OLT is still suitable for osteochondral transplantation because of the continuous effective physical support and sustainable nutritional supply for the repaired surface tissue through the approach. In the current study, we took a cystic depth of OLTs greater than 6 mm as an indication for AOT. On the other hand, patients with OLT areas greater than 144 mm^2^ were excluded to avoid the adverse effect of using multiple bone columns for transplantation.

AOT was mainly previously applied to the surgical treatment of osteochondral lesions of the knee joint [20]. The operation is to repair the defect of the articular cartilage surface by transplanting the autogenous bone-cartilage column into the lesions. At the same time, the subchondral bone in the graft fully fills the cavity and finally integrates with the original bone, providing necessary and continuous nutritional and physical support for the cartilage. The goal of AOT is to implant a graft that is similar in both mechanical and biological properties to that of the patient’s native hyaline cartilage. Several retrospective case series have demonstrated positive results with AOT [8, 16, 21]. Imhoff et al. [22] observed significant long-term improvements in mean AOFAS, VAS, and Tegner activity scores in 26 patients. At a mean follow-up time of 7 years, 18 patients indicated they were very satisfied, 4 satisfied, 3 neutral, and 1 moderately unsatisfied with the procedure. Shimozono et al. [21] systematically reviewed 11 mid-term clinical follow-up studies on AOT treatment for OLTs, including 500 cases in ankle joints, with an average follow-up time of 62.8 months. They found that the average AOFAS ankle and hind foot scores increased from 55.1 preoperatively to 86.2 at the last follow-up, with an excellent rate of 87%. This is similar to the clinical results of the current study reported by us. Autologous grafts are most commonly harvested from the ipsilateral knee, specifically from the lateral femoral condyle or the intercondylar notch. The primary concern with osteochondral autograft transfer is donor site morbidity, as reported by several studies [23]. Shimozono et al. [21] also found that the general incidence of complications after AOT was 10.6%, the most important of which was related to the donor site, and the incidence of donor site morbidity was approximately 3.6%. Fraser et al. [24] reported 40 OLT patients in whom the lateral femoral condyle of the same knee joint was used as the donor site for AOT. After an average follow-up of 24 months, they found that approximately 12.5% of the patients had discomfort symptoms at the donor site, including a painful knee joint and chondromalacia patella during high-intensity exercise. In our study, only 1 case of posterior tibial tendon discomfort caused by the use of too many internal fixation screws after AOT was found. The incidence of complications was 4.3%, which was significantly lower than that reported in previous literature, and there were no donor-site-related complications. This may be related to the selection of the vertical surface of the talus as the donor site. This surgery did not increase the operation time and avoided potential discomfort at the donor site. Also, the osteotomy area may be relative bigger than the oblique osteotomy, but do not increase the bleeding of osteotomy site, and the larger and vertical osteotomy may increase stability and bony union. On the other hand, we used the medial cortical bone of the distal tibia to backfill the talus donor site. The autogenous bone had a strong healing ability, and there was no risk of allogeneic infection. The last follow-up CT scan showed that all the donor-site defects achieved good healing, which also reduced the damage to the stress structure of the talus and the potential risk factors for complications.

The development of arthroscopy technology provided a good choice for minimally invasive treatment of OLTs. However, arthroscopic procedures are mainly used for the cartilage lesions, including debridement, microfracture and BMS. If the damage is deeper than 6 mm, AOT is mostly used to solve the cartilage and bony problems [7]. In this study, the location of OLTs was region IV in 19 patients, region V in 1 patient, and region VII in 3 patients; thus, all the lesions were mainly concentrated in the posterior medial region of the talus where the OLTs usually needed medial malleolar osteotomy for exposure and operation. In general, all kinds of medial malleolar osteotomies not only facilitate the exposure of and operation at the posterior medial area of the talus but also bring additional damage to the medial sensitive area of the ankle joint and the risk of remaining discomfort. Kreuz et al. [25] believed that the total adverse effects of OLT may occur in 3 stages: (1) in the short term, the osteotomy itself can damage adjacent important anatomical structures, such as the posterior tibial tendon, tibial nerve and posterior tibial artery; (2) in the mid-term, the malunion or nonunion caused by poor reduction or fixation of medial malleolus can cause adverse effects; and (3) in the long term, the accelerated degeneration of local articular cartilage can lead to ankle arthritis. Bull et al. [10] reported that the rate of healing-induced deformity after internal malleolar biplane chevron osteotomy was up to 30%. After 2.4 years of follow-up, it was found that approximately 24% of patients had swelling at the osteotomy site, and the residual rate of pain in the internal malleolus was up to 60%. The main reasons were related to poor reduction and internal fixation failure after osteotomy. In the current study, we designed a new medial malleolus osteotomy procedure, a three-plane osteotomy of the medial malleolus, which has the following advantages: (1) By adding the coronal osteotomy of the medial malleolus on the biplane ladder osteotomy, the trauma caused by the osteotomy of the medial malleolus is reduced, the bleeding is reduced, and the adhesion of the posterior medial tendon of the ankle is avoided. (2) By retaining the integrity of the posterior medial bone of the medial malleolus, the risk of injury to important anatomical structures such as the posterior medial neurovascular tendon caused by complete osteotomy of the medial malleolus, such as oblique osteotomy, is avoided. (3) After osteotomy in this way, the osteotomy section at the proximal end of the tibia presents a three-dimensional square concave shape, which is convenient to achieve the complete anastomosis of three planes (the sagittal plane, coronal plane, and horizontal plane) under the direct vision when the distal medial malleolus is restored. In addition, the medial malleolus has high stability after reduction, and reduction is not easily lost during fixation. (4) When hollow screws are used to fix the inner malleolus, vertical compression and firm fixation of screws on three sides is allowed, permitting early functional exercise and weight-bearing activities. In the current study, with the triplane osteotomy method we used, malunion or nonunion of the medial malleolus was not observed, and there was no obvious sign of joint degeneration at the medial malleolar osteotomy site. The average time from osteotomy to full weight-bearing activity was 8.1 weeks, earlier than the medial malleolus healing time reported in the literature [10, 26, 27]. During the last follow-up, the ROM of the affected ankle joint was 12.1° dorsiflexion and 44.7° plantar flexion, and the degree of activity was basically restored to the preoperative level, without obvious ankle joint or tendon adhesion, which may be related to the early active rehabilitation training that we allowed.

There were a few limitations of the present study. First, this was a retrospective study and with all the inherent limitations of a retrospective study. Such as we did not measure the preoperative ROM of ankle joint. Second, this study included a limited number of patients to further conclude the complication or failure relative factors. Third, the follow-up time was relatively short, and the long-term clinical outcomes of this procedure is still unclear, especially the degenerative changes of ankle joint. And, this study did not compare the technique with other medial malleolar osteotomy procedures as a control group, and warrant further investigation.

## Conclusions

These results indicate that AOT combined with medial malleolus triplane osteotomy maybe a viable option for OLTs. Despite the risk of secondary talus injury and internal fixation irritation, the clinical effect and imaging evaluation results at a short follow-up was favorable. The use of the vertical non-weight-bearing area of the talus as the transplantation donor area could reduce the risk of additional surgical injury. Triplane osteotomy of the medial malleolus could reduce the time of osteotomy and trauma, and it was convenient for anatomical reduction and strong internal fixation. However, due to the lack of comparative study with current treatment options, for both the medial malleolus osteotomy and the osteochondral lesion treatments, the advantages of the technique remain indeterminacy. So, the large sample well-designed prospective comparative studies are still needed in the future.

## Data Availability

The data of this study were real and were performed in SPSS 21.0 software (SPSS Inc., Chicago, Illinois). The statistical results of the data are presented in this main paper. The images of case examples are depicted in this research article. All of the data are available in contact with the correspondence author.

## Abbreviations

AOT: Autologous osteochondral transplantation
OLTs: Osteochondral lesions of the talus
BMI: Body mass index
CT: Computed tomography
AOFAS-AH: the American Orthopedic Foot and Ankle Society ankle-hindfoot
VAS: Visual analog scale
ROM: Range of motion
